# Anaphylaxis in an emergency care setting: a one year prospective study in children and adults

**DOI:** 10.1186/s13049-017-0402-0

**Published:** 2017-11-22

**Authors:** Athamaica Ruiz Oropeza, Annmarie Lassen, Susanne Halken, Carsten Bindslev-Jensen, Charlotte G Mortz

**Affiliations:** 1 Department of Dermatology and Allergy Center, Odense Research Center for Anaphylaxis (ORCA), Odense University Hospital, Kløvervænget 15, entrance 142, 5000 Odense, Denmark; 2 Department of Emergency Medicine, Odense University Hospital, Odense, Denmark; 3 Hans Christian Andersen Children’s Hospital, Odense University Hospital, Odense, Denmark

**Keywords:** Anaphylaxis, Epidemiology, Incidence, Prevalence, Symptoms, Treatment

## Abstract

**Background:**

Current data on anaphylaxis is based on retrospective and register based studies. The objective of this study was to describe the epidemiology of anaphylaxis in a 1 year prospective study at the emergency care setting, Odense University Hospital, Denmark (2013–2014).

**Methods:**

Prospective study at the emergency care setting, Odense University Hospital, Denmark (2013–2014). To identify anaphylaxis cases, records from all patients with clinical suspicion on anaphylaxis or a related diagnosis according to the International Classification of Diseases 10 and from patients treated at the emergency care setting or at prehospital level with adrenaline, antihistamines or glucocorticoids were reviewed daily. The identified cases were referred to the Allergy Center, where a standardized interview regarding the anaphylactic reaction was conducted. International guidelines were applied for the assessment of anaphylaxis and its pharmacological treatment. Severity of the anaphylactic reaction was evaluated according to Sampson’s severity score.

**Results:**

We identified 180 anaphylactic patients. Anaphylaxis represented 0.3%–0.4% of all contacts in the emergency care setting with an incidence rate of 26.8 cases per 100,000 person years (95% CI: 14.3–45.8) in children and 40.4 cases per 100,000 person years (95% CI: 32.8–49.3) in adults. Moderate to severe anaphylaxis was registered in 96% of the cases. Skin (96%) and respiratory (79%) symptoms were the most frequent registered, but 7% of cases in adults occurred without skin manifestations. The most common elicitor in children was food (61%), while drugs (48%) and venom (24%) were the main suspected elicitors in adults. Adrenaline was administered in 25% of the cases and it was significantly less administered than glucocorticoids (83%) and antihistamines (91%). The mortality rate during our study period was 0.3 cases per 100,000 person years.

**Discussion:**

This is one of the first prospective studies on the epidemiology of anaphylaxis in children and adults, where the patients are identified not only based on diagnosis codes but also on history, symptoms and treatment and thereafter classified according to international diagnosis criteria for anaphylaxis.

A limitation of this study is that only patients who gave consent to participate in the study were included. Furthermore, patients may have attended other hospitals during the study period. Therefore, the estimates are minimum figures.

**Conclusion:**

The prospective study design with a broad search profile yield a higher incidence than previously reported. Adrenaline was administered in a low proportion of the patients with moderate to severe anaphylaxis. Standardized diagnosis criteria among physicians treating anaphylaxis are needed.

## Background

Anaphylaxis is a severe, life-threatening generalized hypersensitivity reaction [1]. The lifetime prevalence of anaphylaxis in the general population is estimated to be about 0.05–2.0% [2] with a variable Incidence Rate (IR) from 1.5 to 7.9 cases per 100,000 person years in Europe [3] to 42 cases per 100,000 person years in the United States [4]. In the emergency department (ED), anaphylaxis has been reported in 0.04–0.96% of all contacts [5–7]. Overall, the case fatality is reported below 0.0001% [3].

The most severe cases of anaphylaxis are usually related to symptoms from the cardiovascular and respiratory systems [8]. Food has been reported as the most common elicitor in children, while drugs and venom are more frequent in adults [2, 3, 9]). Nevertheless, the diagnosis of anaphylaxis may be difficult in patients with many symptoms, without a certain history of allergy and in the absence of symptoms from the skin and mucosal tissue [1, 10, 11]. Moreover, the elicitors may vary according to age, sex and geographical area [1, 3]. First line treatment of anaphylaxis is adrenaline [1, 12], but previous studies have reported that only a low proportion of anaphylaxis patients receive this treatment [6, 9, 13, 14].

The acute onset of anaphylaxis, the variable symptoms and its transient and unpredictable nature may complicate prospective studies. As a consequence most of the current knowledge on anaphylaxis is derived from retrospective or register based studies [1–4, 6–12]. To our knowledge, only few studies have assessed anaphylaxis prospectively [5, 13, 15]. A retrospective or register based study is likely to underestimate anaphylaxis due to misclassification in diagnosis code, unclear patient history in the ED or the symptoms may have resolved spontaneously, when the patient arrives in the ED or by prehospital treatment. Thus, there is still a lack of evidence as regards the incidence, severity and epidemiological characteristics of anaphylactic reactions.

The aim of this prospective study was to evaluate patients with symptoms, diagnosis and treatment suggestive of anaphylaxis at admission to the emergency care setting. Our primary objective was to estimate the period prevalence and the Incidence Rate (IR) of anaphylaxis during 2013–2014 in children and adults admitted to the emergency care setting of Odense University Hospital. The secondary objective was to describe the severity of the anaphylactic reaction, symptoms, suspected elicitors and the pharmacological treatment administered.

## Methods

We conducted a prospective, non-interventional study in all patients seen at the ED and the Acute Pediatric Ward (APW), Odense University Hospital (OUH), Denmark, during 1^st^ May 2013–30^th^ April 2014.

OUH is a 1000-bed teaching hospital representing all specialties. The population served by the ED and APW consists of four well-defined municipalities with a mixed rural-urban population of 288,587 persons (adults *n* = 240,070; children *n* = 48,517), representative for the general population. The ED attends patients ≥ 15 years of age and the APW children (0–14 years old), with a 24-h acute medical care.

In the prehospital setting, the response to an acute request of prehospital assistance is a two-tiered system, in which an ordinary ambulance manned with two emergency medical technicians (EMTs) is supplied by the Mobile Emergency Care Unit (MECU). The MECU consists of a rapid response car manned with a specialist in Anesthesiology and an EMT [16]. The ambulance can be called directly or the patients can attend either their general practitioners or a doctor on call, and then be referred to the emergency care setting in relevant cases.

Data on the population living in the hospitals catchment area during our study period were collected at the StatBank Denmark website (http://www.statistikbanken.dk; accessed August 2016).

### Participants

**Fig. 1:**
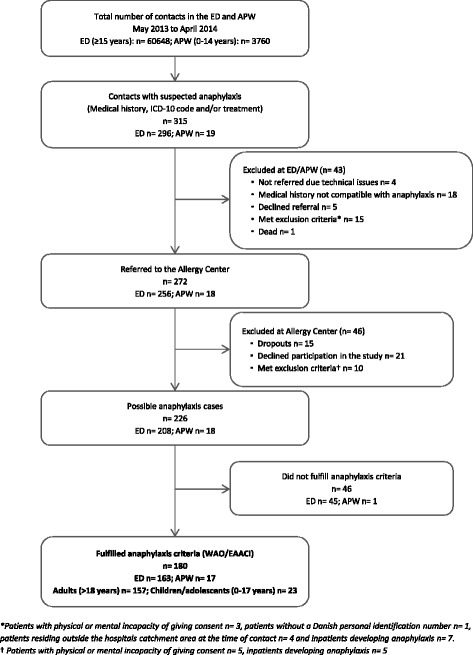
Flowchart of patients with suspected anaphylaxis seen in the Emergency Department (ED) and Acute Pediatric Ward (APW) from May 2013 to April 2014

The cases were eligible when presenting to the ED or APW with any clinical suspicion on anaphylaxis or a diagnosis related to anaphylaxis according to the International Classification of Diseases 10 (ICD-10), and/or when treated at the ED or the APW or at prehospital level with adrenaline, antihistamines or glucocorticoids (Fig. 1).

The eligible cases in the ED were identified by daily electronic screening of the inclusion criteria among all records in the ED. All the matched records were reviewed to recognize the possible anaphylaxis cases. To identify the eligible cases from the APW, all records for the admitted patients during the study period were reviewed, and diagnose codes for children observed during admission for less than 24 h were reviewed. Furthermore, all medical doctors in the ED and APW were informed to refer all patients with suspicion of anaphylaxis.

The identified cases were referred to the Allergy Center (AC) and assessed according to the World Allergy Organization (WAO) [1] and the European Academy of Allergy and Clinical Immunology (EAACI) [12] clinical criteria for the diagnosis of anaphylaxis (Fig. 1). These criteria state that anaphylaxis is highly likely when any of the following 3 criteria is fulfilled: 1) sudden skin and/or mucosal tissue symptoms together with respiratory compromise and/or severe hypotension (systolic pressure under 90 mmHg for adults and age-specific for children) and/or end-organ dysfunction, 2) symptoms from two or more organ systems (skin and/or mucosal tissue, respiratory, cardiovascular and/or gastrointestinal systems) suddenly after exposure to a likely allergen, or 3) severe hypotension after exposure to a known allergen for that patient. Only patients fulfilling WAO/EAACI clinical criteria for the diagnosis of anaphylaxis were included.

Patients with physical or mental incapacity of giving consent, without a Danish personal identification number and/or residing outside the hospitals catchment area at the time of contact were excluded, as well as inpatients developing anaphylaxis (Fig. 1). If a patient had multiple contacts with anaphylaxis at the ED or the APW over the study period, data from the first contact was used.

### Data collection

During the first visit at the AC a standardized interview regarding symptoms, suspected elicitors and administered treatment in relation to the anaphylactic reaction was conducted. The history, objective findings including vital parameters, and the administered pharmacological treatment in the ED, APW and at prehospital level were drawn and complemented from the patient files. Data were entered in duplicate in an EpiData® database.

### Outcomes

The period prevalence of anaphylaxis cases at the ED and APW was calculated as the number of included children and adolescents/adults per total number of contacts in the APW and the ED, respectively. The period prevalence in the population was calculated as the number of patients having anaphylaxis per the total numbers of habitants in the hospital’s catchments area during the study period. Finally, the IR of anaphylaxis was calculated as the number of patients having anaphylaxis for the first time per 100,000 person years.

Although adolescents (15–17 years) were attended in the ED at OUH, the variable age group was defined as children/adolescents (0–17 years) and adults (>18 years). Age was given as median and 25–75 percentiles (IQR).

The severity of the anaphylactic reaction was graduated according to Sampson’s severity score [17], the severity score that is used routinely in our AC [18]. It consists of five severity grades; from grade 1, mild symptoms, to grade 5 severe symptoms. The severity grade depends on symptoms from the organ system that is mostly affected. In this scoring system is possible to graduate the reaction also if only single symptoms, such as hypotension, are registered, and it refers to key symptoms that are absolute indications for adrenaline administration. Moderate to severe anaphylaxis was defined as grade 3–5.

The pharmacological treatment administered was assessed in relation to EAACI guidelines recommendations [12]. First-line: adrenaline intramuscular (IM) or intravenous (IV). Third-line: antihistamines and glucocorticoids. Inhaled adrenaline was also assessed. Additionally, we evaluated the administration of adrenaline according to recommendations in Sampson’s severity score and the prescription of adrenaline auto injector at the discharge point.

### Statistical analysis

Statistical analysis was performed with Stata IC 14.0 (Stata Corporation LP®, College Station, Texas, USA). Comparisons were made by *χ* ^2^-based table analysis. Statistical significance was defined as *p < 0.05.* Both the IR and the period prevalence were calculated with the correspondent 95% confidence interval based on a Poisson distribution (CI 95%).

### Ethics

The study was approved by the Data Protection Agency (J. no. 12/26172) and the Regional Committees on Health Research Ethics for Southern Denmark (J. no. S-20120203). The patients were included after informed consent.

## Results

Numbers of individuals at each stage of the study and reasons for exclusion are presented in Fig. 1. Of these, 180 (female 55%, male 45%) fulfilled the criteria for the diagnosis of anaphylaxis and were included in the study; 157 (87%) adults with a median age of 45 years (IQR, 31.5–60.5) and 23 (13%) children/adolescents with a median age of 9 years (IQR, 2–15).

The proportion of individual patients with anaphylaxis in the APW was calculated to 0.4% (95% CI: 0.2–0.7) of all contacts and in the ED to 0.3% (95% CI: 0.2–0.3) of all contacts. The period prevalence of anaphylaxis in OUH’s catchments area was estimated to 0.04% (95% CI: 0.03–0.07) in children and 0.06% (95% CI: 0.05–0.07) in adults. Fifteen patients (children *n* = 3, adults *n* = 12) were seen more than once during the study period representing eighteen extra contacts in the emergency care setting.

Among the 180 patients included in the study, 110 (children *n* = 13, adults *n* = 97) experienced anaphylaxis for the first time giving an IR of 26.8 cases per 100,000 person years (95% CI: 14.3–45.8) in children and 40.4 cases per 100,000 person years (95% CI: 32.8–49.3) in adults.

One fatality due to anaphylaxis was registered during the study period. This fatal case represented 0.5% of our study population and a mortality rate of 0.3 cases per 100,000 person years in our study period. Since the patient died before arriving to the ED we chose to describe the findings independently and therefore this case is not included in further analysis.

### Severity of the anaphylactic reaction and symptoms

**Fig. 2:**
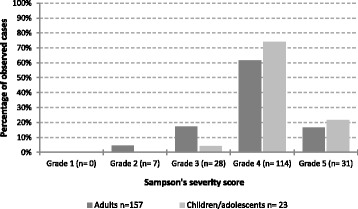
Severity grades of anaphylaxis (Sampson’s severity score) in children/adolescents and adults fulfilling the WAO/EAACI criteria for the diagnosis of anaphylaxis (*n* = 180)

None of those fulfilling the WAO/EAACI criteria for anaphylaxis had mild anaphylaxis according to Sampson’s severity score (grade 1). Only 7 cases had grade 2 anaphylaxis, while 173 (96%) had moderate to severe anaphylaxis (grade 3–5) (Fig. 2). There was no statistical difference in the severity of anaphylaxis among children/adolescents compared to adults.

Skin manifestations and respiratory symptoms were the most frequent registered symptoms (Table 1). A total of 169 patients (94%) had skin symptoms and 143 (79%) had respiratory symptoms. There was no statistical difference in the distribution of the symptoms from the different organ systems when comparing children/adolescents with adults neither when including all patients with anaphylaxis nor in the group with moderate to severe anaphylaxis. However, in the group of patients with grade 4–5 anaphylaxis, cardiovascular symptoms were more often reported in adults than in children/adolescents (*p = 0.056*) with tachycardia and severe hypotension as main symptoms.

**Table 1:** Symptoms listed by organ system according to WAO anaphylaxis guidelines [1] comparing children/adolescents and adults fulfilling the WAO/EAACI criteria for the diagnosis of anaphylaxis (*n* = 180)

Symptoms	Children/adolescents *n* = 23 (%)	Adults *n* = 157 (%)
**Skin, subcutaneous tissue and mucosa**	**23 (100)**	**146 (93)**
Generalized itching	11 (48)	69 (47)
Generalized flushing/erythema	12 (52)	63 (43)
Generalized urticaria	7 (30)	53 (36)
Localized angioedema	7 (30)	55 (38)
Generalized angioedema	9 (39)	46 (32)
**Respiratory tract**	**19 (83)**	**124 (79)**
Sensation of throat tightness	7 (37)	78 (63)
Throat itching	7 (37)	27 (22)
Hoarseness	6 (32)	30 (24)
Wheezing/bronchospasm	11 (58)	48 (39)
**Gastrointestinal tract**	**14 (61)**	**95 (61)**
Dysphagia	5 (36)	47 (49)
Abdominal pain	9 (64)	30 (32)
Nausea	9 (64)	52 (55)
Vomiting	5 (36)	21 (22)
Diarrhea	3 (21)	13 (14)
**Cardiovascular system**	**10 (43)**	**94 (60)**
Tachycardia^a^	7 (70)	71 (76)
Mild Hypotension^b^	1 (10)	10 (11)
Severe hypotension^c^	1 (10)	15 (16)
Urinary or fecal incontinence	1 (10)	7 (7)
**Central Nervous System**	**11 (48)**	**72 (47)**
Uneasiness	1 (9)	10 (14)
Altered mental status	7 (64)	42 (58)
Dizziness	0 (0)	12 (17)
Loss of consciousness	3 (27)	15 (21)

^a^Heart rate above or equal to 100 beats per minute

^b^ Systolic blood pressure between 110 and 90 mmHg according to references [1, 12]

^c^ Systolic blood pressure below 90 mmHg according to references [1, 12]

Boldface are marking the 5 different organ systems and for each organ system the percentage distribution of symptoms are given

### Elicitor profile

Drugs (44%), food products (22%) and venom (21%) were the most frequent reported elicitors in our study group. The elicitor profile varied according to the age groups; food products were more often reported in children/adolescents compared to adults (*p < 0.001*), while drugs (*p = 0.019*) and venom (*p = 0.035*) were more often registered in adults compared to children. In 12% of all cases the elicitors were unknown. Inhalation allergens (cat) were reported in only 1% of the patients (Table 2).

**Table 2:** Suspected elicitors of anaphylaxis in children/adolescents and adults fulfilling the WAO/EAACI criteria for the diagnosis of anaphylaxis (*n* = 180)

Suspected elicitors	Children/adolescents *n* = 23 (%)	Adults *n* = 157 (%)	*p value*
**Drugs**	**5 (22)**	**75 (48)**	**0.019**
β-lactam antibiotic	1 (20)	30 (40)	
ACE inhibitors	0	13 (17)	
ASA/NSAIDs	3 (60)	9 (12)	
Other drugs^a^	1 (20)	23 (31)	
**Food**	**14 (61**)	**26 (17)**	**<0.001**
Tree nuts	7 (50)	8 (31)	
Peanuts	3 (21)	1 (4)	
Fish/shellfish	0	3 (11)	
Wheat	0	3 (11)	
Other foods^b^	4 (29)	11 (42)	
**Venom**	**1 (4)**	**37 (24)**	**0.035**
Wasp	1 (100)	25 (67)	
Bee	0	8 (22)	
Other insects^c^	0	4 (11)	
**Inhalation allergens**	**1 (4)**	**1 (1)**	**0.113**
**Unknown**	**2 (9)**	**18 (11)**	**0.693**

^a^ Iodinated Radio Contrast Media *n* = 5, Macrolides *n* = 1, Miconazole *n* = 1, Acyclovir *n* = 1, Chloramphenicol *n* = 1, Metronidazole *n* = 2, Proton pump inhibitor *n* = 1, Acrivastine *n* = 1, Chlorhexidine *n* = 1, Gabapentin *n* = 1, Camphorated opium tincture *n* = 1, Xylometazoline *n* = 1, Lamotrigine *n* = 1, topic Glucocorticoids *n* = 1, Calcium antagonist (felodipine) *n* = 1, Mesalazine *n* = 1, Valerian root *n* = 1, Zopiclone *n* = 1, D- vitamin *n* = 1

^b^ Milk *n* = 1, Carrot *n* = 1, Celery *n* = 1, Bugles chips *n* = 1, Oatmeal breakfast cereal *n* = 1, Tomato *n* = 1, Fruit/fig bars (apple/raspberry) *n* = 1, Kiwi, parsley root and parsnip *n* = 1, Poppy seed *n* = 1, Carry *n* = 1 Chicken, bread, pasta, sour cream *n* = 1, Dressing (hydrolysate) *n* = 1, Red wine *n* = 1, Jensens Bøfhus® sauce *n* = 1, Noodle soup *n* = 1

^c^ Bumblebee, gadfly

Boldface are marking the different groups of elicitors and for each group the percentage distribution of the specific elicitors are given

### Treatment

Independently of the severity of the reaction, glucocorticoids and antihistamines were more often administrated than adrenaline.

Of the 180 patients included 37% (*n* = 66) received medical attention at prehospital level (General Practitioner *n* = 6, Doctor on call *n* = 7, MECU *n* = 48 and from both General Practitioner and MECU *n* = 5).

Treatment with adrenaline was mostly administered at prehospital level, while glucocorticoids and antihistamines were more frequently administrated at the ED and APW (Table 3).

**Table 3:** Applied drugs among patients fulfilling the WAO/EAACI criteria for the diagnosis of anaphylaxis. Several answers are possible (*n* = 180)

Treatment	Pre-hospital			Hospital	Total cases *n* = 180 (%)
	Bystander^a^	Physician^b^	Ambulance/MECU	Emergency Department	
**Adrenaline**					**45 (25)**
IM	10	4	23	7	44 (24)
IV	0	0	2	1	3 (2)
**Glucocorticoids**					**150 (83)**
IV	1	8	36	87	132 (73)
Oral	4	1	0	20	25 (14)
**Antihistamines**					**164 (91)**
IV	1	8	41	86	136 (76)
Oral	45	8	0	17	70 (39)
**Adrenaline Inhalation** ^c^	0	0	9	7	**16 (9)**

^a^ Patient/relatives, Personal in institution or at work or Friend

^b^ General practitioner or doctor on call

^c^4/16 patients received also adrenaline IM

Boldface are marking the different treatments and the percentage distribution, including administration way, are given as percent of the total number of cases

**Fig. 3:**
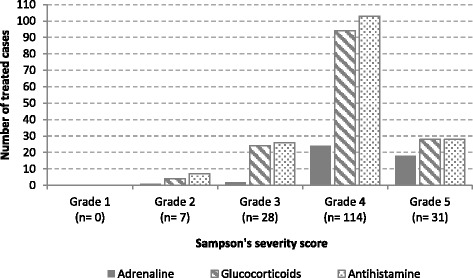
Treatment administrated in relation to the severity of the anaphylactic reaction in patients fulfilling WAO/EAACI criteria for the diagnosis of anaphylaxis (*n* = 180)

In the group of patients with moderate to severe anaphylaxis (grade 3–5) only 25% (44/173) received treatment with adrenaline according to guidelines (IM 93%, IV 2%, IM + IV 5%) compared to the 84% (146/173) receiving glucocorticoids (*p < 0.001*) and the 91% (157/173) receiving antihistamines (*p < 0.001*). Even in grade 5, only 58% (18/31) were treated with adrenaline (Fig. 3).

Adrenaline inhalations were administered in 9% (16/173) of the patients with moderate to severe anaphylaxis, of these 25% (4/16) were treated concomitantly with IM adrenaline.

Based on symptoms in Sampson’s severity score adrenaline should be administered immediately in 85% (147/173) of the cases in grade 3–5; of those only 28% (41/147) received adrenaline.

When comparing administered treatment in the group of children and adults separately, the results did not change.

Adrenaline auto injector was prescribed at the discharge from the APW and ED in 40% of patients with food as the suspected elicitor of anaphylaxis and in 60% of those with venom as the suspected elicitor.

## Discussion

This is one of the first prospective studies on the epidemiology of anaphylaxis in children and adults at a large university hospital, where the patients are identified not only based on diagnosis code but also on history, symptoms and treatment and thereafter classified according to international diagnosis criteria for anaphylaxis.

We found that the proportion of anaphylaxis in the emergency setting is in line with previous epidemiological studies in the ED [5, 6, 14] and the APW [7]. Moreover, the result suggests that in the group of adults, anaphylaxis presentation in the ED is as frequent as ST-elevation myocardial infarction [19].

In contrast, we found a higher IR of anaphylaxis (40.4 cases per 100,000 person years), compared to previous studies in Europe, where the IR is reported to be 1.5–7.9 cases per 100,000 person year [3], and in Denmark where the IR is reported to be increasing from 3.2 cases per 100,000 person year late in the 80’s [20] to 6.46 cases per 100,000 person year during 1995–2012 [21]. The difference could be explained by different inclusion criteria in the studies, and more specific the diagnosis codes included. In both the retrospective and register based studies of Soerensen et al [20] and Jeppesen et al [21], only the cases having diagnosis codes related to “anaphylactic shock” were included. In our study, records from 42 allergy related diagnoses (ICD-10) were reviewed, as well as all records with administered treatment of adrenaline, antihistamines or glucocorticoids to find as many cases as possible. Finally, the WAO/EAACI criteria were used for our final diagnosis of anaphylaxis. Similar differences were previously described by Bohlke [22], who could identify six times more anaphylactic episodes among children and adolescents between 1991–1997, when incorporating other allergy diagnosis besides the diagnosis codes specific for anaphylaxis. In the same way, a new retrospective and register based study of Lee et al [4] including many anaphylaxis related diagnosis (ICD-9), reported also a high IR of anaphylaxis in 42 cases per 100,000 person year, in line with our results.

Concerning the clinical characteristics of anaphylaxis, our results are in line with previous studies: the high proportion of moderate to severe anaphylaxis cases regardless of the severity score applied [9, 13],skin and respiratory symptoms as the most prevalent symptoms [5, 14, 23] and cardiovascular symptoms being more prevalent among adults compared to children [11]. It is important to highlight that 7% of the adults in our study population did not have symptoms from the skin and mucosal tissue, whereas previous studies described the absence of skin symptoms in up to 20% of anaphylaxis cases [1, 10, 11]. The latter may represent a diagnostic challenge, especially when it is not possible to collect an appropriate history of symptoms or elicitors as it may be the case with children, elderly or unconsciousness patients.

Age related differences in the elicitors profile have also been described in other studies in Europe and North America [2, 3, 7, 9]. These differences could be explained by the fact that adults are more exposed to drugs and insect stings than children/adolescents. Furthermore, food allergies are more common in children and the prevalence decreases with increasing age [24, 25].

Finally, our results about treatment of anaphylaxis substantiate those from others studies worldwide, where glucocorticoids and antihistamines seems to be administered almost as a routine, and adrenaline is administered in a lower proportion [6, 9, 13, 14, 23]. This is in contrast to international guidelines [1, 12]. Adrenaline as the first-line drug for anaphylaxis was administered in only 25% of the 173 patients with moderate to severe anaphylaxis in our study group. Whether these findings reflect the assessment of the physician on charge or insufficient treatment of the anaphylactic reaction or both cannot be clarified. However, the low degree of administration of adrenaline may be related to various factors. Firstly, the fact that anaphylaxis is mostly a prehospital condition with rapid onset, where the only possibilities to get the treatment immediately is, that the patient is equipped with an adrenaline auto injector (as was the case for 10 of our patients) or that the patient get medical help immediately (as 66 of our patients did, but only 27 were treated with adrenaline). This can probably explain why adrenaline was mostly administered at prehospital level in our study. Secondly, the improvement or even disappearance of the symptoms may occur spontaneously in some cases or because the allergen in question is removed (as may be the case in food induced anaphylaxis after vomiting). Besides, in some cases pre-existing cardiovascular conditions may have affected the decision on whether to use epinephrine or not.

Adrenaline is recommended to treat anaphylaxis due to its effect on α-1, β-1 and β-2 receptors [12] and is the only drug effective for all symptoms in anaphylaxis including hypotension and the severe respiratory symptoms [12]. The progression is fast, the course unpredictable and delayed injection of adrenaline has been described to be associated with fatal anaphylaxis [26, 27]. These facts support the importance of the prompt administration of adrenaline as well as the prescription of adrenaline auto injectors at the discharge from the emergency care settings and the reference of the patients to further allergological investigation. Standardized educational interventions including treatment algorithm and lectures on recognition, grading and management of anaphylaxis among personal at prehospital level and the staff at the ED and APW, could improve the timely administration of adrenaline.

A limitation of this study is that only patients who were referred to the Allergy Center and gave consent to participate in the study were included. Therefore, our estimates are minimum figures as we cannot rule out that some of the patients who declined participation, or were excluded, could have had anaphylaxis. Furthermore, some contacts may have been missed since some patients living in OUH’s catchments area may have attended other hospitals during the study period for anaphylaxis. Also, a potential source of error is the possibility of cases of fatal anaphylaxis at pre-hospital level not being registered in the MECU or ED. Finally, the elicitor profile is based on the patient history rather than on diagnostic work-up.

The strengths of our study are the prospective inclusion of the patients with a broad search on possible anaphylaxis cases comprising the patient history, the administered treatment and a wide list of possible IDC-10 diagnoses related to allergy and anaphylaxis. Besides this, our study is based in a population that is representative for the Danish general population. Furthermore, due to a carefully interview at the Allergy Center and use of patients files from the ED and APW, it was possible to classify all patients according to established diagnostic criteria of anaphylaxis and a severity score.

In this study focus was on the assessment and management of anaphylaxis at the admission to the emergency care setting. In the future, this patient cohort will be followed up after allergological diagnostic work-up to verify the elicitors along with evaluation of co-morbidity and co-factors in anaphylaxis.

## Conclusion

Anaphylaxis occurred with a higher incidence compared to previous studies but with the same elicitor profile. Skin and respiratory symptoms were the most frequently observed, but up to 7% of cases in adults occurred without skin manifestations. The main elicitor of anaphylaxis in children was food, while in adults drugs and venom were the main elicitors. Adrenaline was administered significant less than glucocorticoids and antihistamines. Our findings reflect the need for standardized anaphylaxis criteria among physicians treating anaphylaxis as well as the implementation of anaphylaxis guidelines regarding diagnosis and treatment.

## Abbreviations

AC: Allergy center
APW: Acute pediatric ward
EAACI: European academy of allergy and clinical immunology
ED: Emergency department
EMTs: Emergency medical technicians
ICD-10: International classification of diseases 10
IM: Intramuscular
IR: Incidence rate
IV: Intravenous
MECU: Mobile emergency care unit
OUH: Odense university hospital
WAO: World Allergy Organization
